# A Method for Analysis of Free and Total Ropivacaine in Dog Plasma Using UHPLC–MS/MS

**DOI:** 10.1002/bmc.70214

**Published:** 2025-09-03

**Authors:** Natali Verdier, Karin Hummel, Ebrahim Razzazi‐Fazeli

**Affiliations:** ^1^ Anaesthesia and Perioperative Intensive Care, Clinical Centre for Small Animal Health and Research, Clinical Department for Small Animals and Horses University of Veterinary Medicine Vienna Vienna Austria; ^2^ VetCore Facility (Mass Spectrometry) University of Veterinary Medicine Vienna Vienna Austria

**Keywords:** liquid chromatography, mass spectrometry, method validation, rapid equilibrium dialysis, ropivacaine

## Abstract

Ropivacaine is a local anesthetic commonly used in veterinary anesthesia. A liquid chromatography–mass spectrometry (LC–MS) method was developed to quantify free and total ropivacaine in dog plasma, which included rapid equilibrium dialysis. The method was validated for selectivity, specificity, matrix effect, calibration curve and range, accuracy and precision, carry‐over, stability, and reinjection reproducibility according to the International Conference on Harmonization M10 guidelines. After ultra‐high performance liquid chromatographic (UHPLC) separation, detection and quantification of ropivacaine was performed using a triple quadrupole tandem mass spectrometer with electrospray ionization. LC–MS method validation was carried out in a range of 0.05–1000 ng/mL ropivacaine in dog plasma in two dilutions (1:1 and 1:4). The precision and accuracy of the method were determined at four concentration levels and ranged from 0.40% to 5.30% and 85.50% to 113.30%, respectively. The lower limit of quantification was as low as 0.30 and 0.05 ng/mL, for the quantitation of protein‐bound (1:4) and free (1:1) ropivacaine, respectively. All validation parameters met acceptance criteria. This UHPLC–MS/MS method was successfully applied in a clinical study that involved the intraperitoneal instillation of ropivacaine to anesthetized dogs and can be used to quantify free and total ropivacaine in dog plasma.

## 1 Introduction

Ropivacaine is a well‐established amide‐type local anesthetic, commonly used to provide analgesia both during and after painful surgical procedures in animals (Grubb and Lobprise 2020). As with other local anesthetics, ropivacaine reversibly blocks the conduction of nerve impulses by inhibition of voltage‐gated sodium channels (Butterworth and Strichartz 1990). The lipid solubility of ropivacaine is intermediate between lidocaine and bupivacaine, and the plasma protein binding, mainly to alpha‐1 acid glycoprotein, is 94% in humans and 99% in dogs (Feldman et al. 1996; McClure 2002; Thomas and Schug 1999). Ropivacaine is advantageous over other local anesthetics because it has a longer duration of action than lidocaine and is less cardio‐and neurotoxic than bupivacaine (Dony et al. 2000; Feldman et al. 1989; McClure 2002).

Intraperitoneal instillation of local anesthetics is an extensively studied and simple regional anesthetic technique used to provide analgesia for abdominal surgery in many species (Benito et al. 2016; Goldstein et al. 2000; Lambertini et al. 2018). However, using recommended doses of ropivacaine, studies in dogs demonstrate only a partial benefit in the post‐operative period (Gomes et al. 2020; Kazmir‐Lysak et al. 2023; Lambertini et al. 2018). The pharmacokinetic profile of ropivacaine after intraperitoneal instillation has been studied in humans (Labaille et al. 2002) and pigs (Betton et al. 2010). However, there are currently no pharmacokinetic studies of ropivacaine after intraperitoneal instillation in dogs, which would be necessary to further characterize the pharmacokinetic profile of this drug and eventually determine if an increase in dose would result in a better analgesic efficacy.

Several methods have been described to quantify ropivacaine in different matrices and species (Cui et al. 2023; Engman et al. 1998; Sawaki et al. 2009). Of these, only a few have developed a method to determine the free and total ropivacaine in human plasma (Abbas et al. 2013; Lamy et al. 2020; Mathieu et al. 2006). Although one study reported the plasma protein binding of ropivacaine in dogs (Feldman et al. 1996), there is, to date, no validated bioanalytical method published for the quantification of free and total ropivacaine in dog plasma. To address this gap, we have developed and validated a highly sensitive ultra‐high performance liquid chromatography–mass spectrometry (UHPLC–MS/MS) method with a lower limit of quantification (LLOQ) of 0.05 and 0.30 ng/mL for free and protein‐bound ropivacaine in dog plasma, respectively. The method developed was based on a previously published method to determine free and total ropivacaine in human plasma that followed the guidelines of European and American regulatory agencies (Lamy et al. 2020). By providing a new, highly sensitive method for quantifying free and total ropivacaine in dog plasma, this work may serve as a basis for future studies on the drug's pharmacokinetics and analgesic efficacy.

## 2 Material and Methods

### 2.1 Chemicals and Reagents

Ropivacaine [(2S)‐N‐(2,6‐dimethylphenyl)‐1‐propyl‐2‐piperidinecarboxamide, monohydrochloride, monohydrate)] and the internal standard (IS) D7‐ropivacaine [(S)‐N‐(2,6‐dimethylphenyl)‐1‐(propyl‐d_7_)piperidine‐2‐carboxamide, monohydrochloride)] were obtained from Cayman Chemical (Michigan, USA). Formic acid (99%) and water, both Optima LC–MS grade, were purchased from Fisher Scientific (Waltham, MA, USA). Acetonitrile (hypergrade for LC–MS LiChrosolv) was supplied by Merck (Darmstadt, Germany). Phosphate buffered saline (PBS) tablets (Rotifair PBS 7.4) were obtained from Roth (Karlsruhe, Germany). Blank dog plasma samples were sourced from remnants provided by the Institution's (University of Veterinary Medicine Vienna) Laboratory. All other chemicals were of analytical grade or higher.

### 2.2 Preparation of Calibration and Spike Standards

Stock solutions of ropivacaine and the deuterium‐labelled IS D7‐ropivacaine (1 g/L) were prepared in water. Working solutions of ropivacaine were prepared with water at different concentrations (50, 10, and 2.5 μg/mL) as spike solutions for complete workflow validation, and at 10 μg/mL for dilution of the standard curve as well as for blank matrix spiking for the 0.05, 0.15, 0.3, 1, 300, and 750 ng/mL QC levels for the LC–MS method validation procedure. Working solutions of IS (10 and 100 ng/mL) were obtained by dilution of the D7‐ropivacaine stock solution with water. Stock and working solutions were stored at −20°C.

### 2.3 Instrumentation

An Agilent 1290 Infinity II (Agilent Technologies, Santa Clara, USA) Multisampler (G7167B, Agilent Technologies) and a Highspeed Pump (G7120A, Agilent Technologies) were used as a chromatographic UHPLC system. Compound detection and quantification were performed using a QTRAP 6500+ mass spectrometer (AB Sciex, MA, USA) in multiple reaction monitoring (MRM) mode. SciexOS software (Version 3.1.0.16485) was used for data acquisition, processing, and quantification.

### 2.4 Sample Preparation With Rapid Equilibrium Dialysis (RED)

**FIGURE 1:**
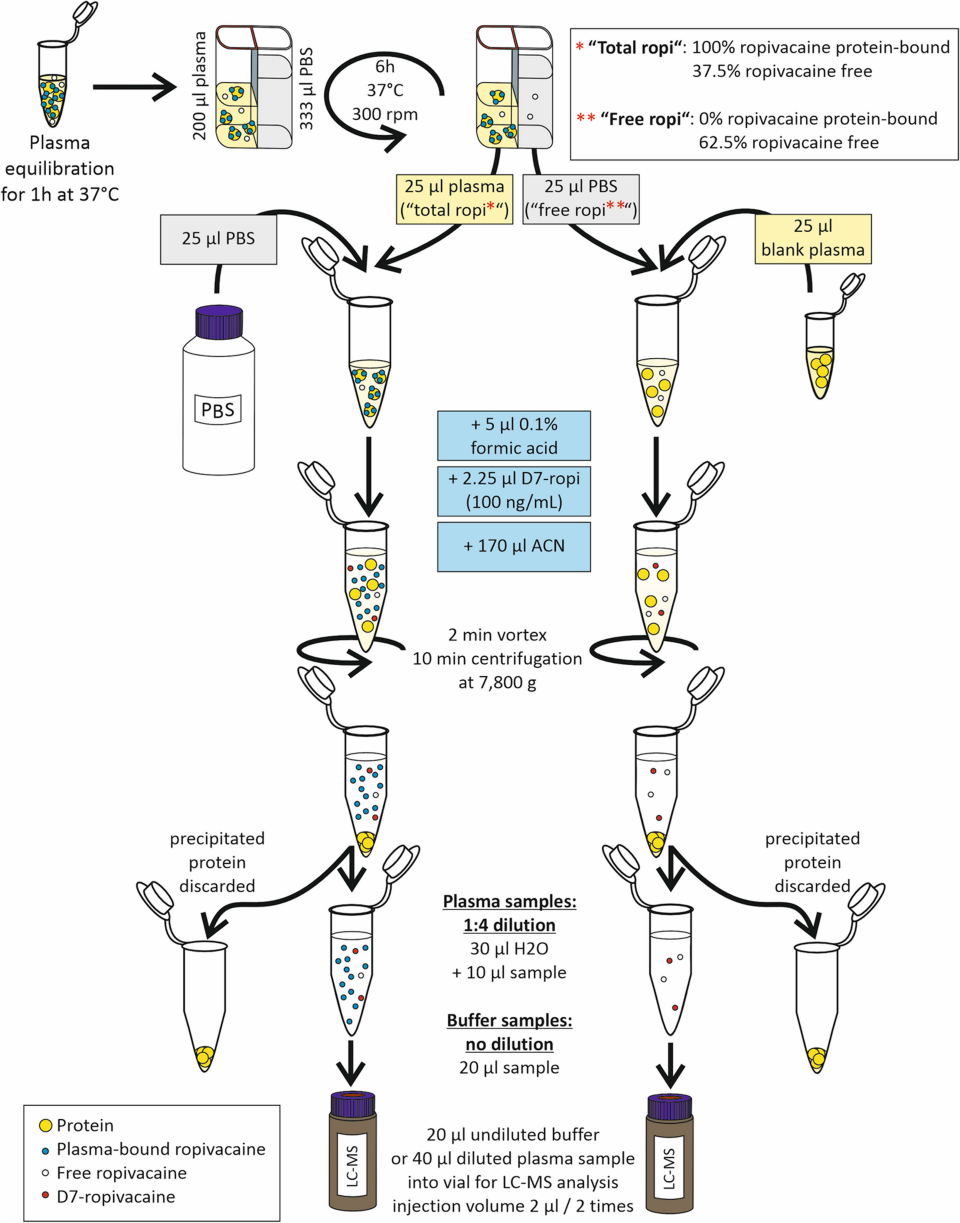
Sample preparation scheme including rapid equilibrium dialysis.

For generation of blank plasma, complete workflow validation, and study sample preparation, dog plasma (blank or spiked) was temperature equilibrated for 1 h at 37°C in an Eppendorf thermomixer (Hamburg, Germany) (Figure 1). The free and protein‐bound ropivacaine fractions were separated using an RED device, with plates containing single‐use inserts made of two side‐by‐side chambers, separated by a vertical cylinder of an 8 kDa molecular weight cut‐off dialysis membrane (Fisher Scientific, Vienna, Austria).

Each insert was filled with 200 μL of plasma sample on one side and 333 μL of PBS buffer on the other side. The support plate of the dialysis device was covered with a self‐adhesive plastic film. Dialysis was carried out for 6 h at 37°C with shaking at 300 rpm. Then, 25 μL were taken from the plasma (representing protein‐bound ropivacaine plus 37.5% of the absolute amount of free ropivacaine) and buffer (representing 62.5% of the absolute amount of free ropivacaine) compartments and transferred separately to conical 1.5 mL tubes. Then, 25 μL of blank plasma was added to the buffer sample and 25 μL of PBS buffer was added to the plasma sample in order to reach a homogenous matrix background. After acidification with 5 μL 0.1% aqueous formic acid and the addition of 2.25 μL of 100 ng/mL IS (D7‐ropivacaine), samples were vortexed. For protein precipitation, 170 μL of acetonitrile was added. Samples were vortexed for 2 min and centrifuged at 7800*g* for 10 min. Subsequently, supernatants were transferred to fresh tubes. Prior to injection into the LC–MS system, plasma fractions were diluted 1:4 with water, whereas buffer fractions were injected undiluted in order to compensate for the concentration differences between protein‐bound and free ropivacaine.

Calculation of the concentration of free and protein‐bound ropivacaine in the original plasma sample was performed using the formulas in Supporting Information S1: Appendix A.

### 2.5 Liquid Chromatography and Mass Spectrometer Settings

Two microliters of samples and standards were injected into the LC–MS system. Chromatographic separation was performed using a 1.7 μm Kinetex F5 100 Å column (100 × 2.1 mm inner diameter) (Phenomenex Inc., Torrance, USA) maintained at 40°C in a Multicolumn Thermostat (G7116B, Agilent Technologies). The flow rate was set at 400 μL/min and the total run time was 9 min. The mobile phase was a gradient of water (A) and acetonitrile (B) both containing 0.1% formic acid. It started with 10% B and increased to 50% within 4 min. Thereafter, within 0.5 min, mobile phase B was increased to 90% and held for 2 min for column washing. Conditions were changed back to 10% B within 0.5 min and kept for 2 min at starting conditions for column equilibration.

Electrospray ionization in positive mode was performed using a Turbo V ion source (AB Sciex). Scanning of precursor and fragment ions of ropivacaine and the IS D7‐ropivacaine for the development of the MRM acquisition method as well as compound parameter optimization was performed by direct infusion of the standards with a syringe pump with a flow rate of 5 μL/min and a concentration of 10 ng/mL in 50% acetonitrile/50% H_2_O. The MRM transitions used as quantifiers were set to m/z 275.1 → 84 Da and m/z 282.1 → 85.1 Da for ropivacaine and D7‐ropivacaine, respectively, whereas m/z 275.1 → 98.2 Da and m/z 282.1 → 105.1 Da were used as qualifiers for ropivacaine and D7‐ropivacaine (Table 1). Subsequently, source parameters were optimized by flow injection analysis (FIA). The resulting source parameters were as follows: curtain gas 35 psi; ion spray voltage 3500 V; source temperature 550°C; ion source gas 1 and 2 were 60 psi; CAD gas set to 9.

**TABLE 1:** Compound parameters QTRAP 6500+.

Compound ID^a^	Q1 mass (Da)	Q3 mass (Da)	Collision energy (ce) (V)	Collision exit cell potential (CXP) (V)
Ropivacaine	275.1	84.0	59	12
Ropivacaine_qual	275.1	98.2	55	12
D7‐ropivacaine	282.1	85.1	57	10
D7‐ropivacaine_qual	282.1	105.1	55	12

^a^ Dwell time constantly 50 ms, entrance potential (EP) 10 V, and declustering potential (DP) 85 V for all transitions.

### 2.6 LC–MS Method Validation Procedure

A method for the quantification of free and total ropivacaine in dog plasma was developed according to Lamy et al. (2020) and fully validated for selectivity, specificity, matrix effect, calibration curve and range, accuracy and precision, carry‐over, stability, and reinjection reproducibility according to the International Conference on Harmonization M10 guidelines on bioanalytical method validation and study sample analysis (European Medicines Agency 2022).

#### 2.6.1 Selectivity and Specificity

The selectivity and specificity of the method were evaluated by using blank plasma (non‐lipaemic and non‐hemolyzed) samples from six dogs to ensure the absence of interfering substances. Selectivity and specificity were accepted if the interfering signal was equal to or less than 20% of the response at LLOQ for ropivacaine and 5% for the IS.

#### 2.6.2 Matrix Effect

For assessment of the matrix effect, three replicates of low and high quality controls (QCs) were used, each prepared using matrix from six different dogs. For the matrix effect to be acceptable, the accuracy of each sample had to be within ±15% of the nominal concentration and the precision (%CV) of each sample had to be lower than 15%.

#### 2.6.3 Calibration Curve and Range

The linear relationship between analyte concentration and response (area ropivacaine/area D7‐ropivacaine) was evaluated and confirmed separately for each LC–MS acquisition run. Two separate standard curves were prepared: For plasma samples containing protein‐bound ropivacaine and therefore higher ropivacaine concentrations, the standards were 1:4 diluted with water. For the buffer samples containing only free ropivacaine and therefore very low ropivacaine concentrations, the standard curves were prepared in pure blank matrix. To evaluate the linearity of the method, calibration curves were generated using 9 and 13 concentration levels of calibration standard for the 1:1 and 1:4 dilution, respectively, that included the LLOQ and the upper limit of quantification (ULOQ). The concentration levels were 0.05, 1, 10, 100, 200, 400, 600, 800, and 1000 ng/mL for the 1:1 dilution and 0.075, 0.1, 0.25, 0.3, 0.5, 1, 10, 100, 200, 400, 600, 800, and 1000 ng/mL for the 1:4 dilution. LC–MS data of the calibration curve were acquired in technical triplicates. Linear regression was calculated weighting by 1/x in SciexOS software. A correlation coefficient (*R* ^2^) greater than 0.99 was required for each calibration curve to be acceptable. Back‐calculations were made from the curve equations to determine the concentration of each analyte in each individual calibration standard sample. The accuracy of the back‐calculated concentrations of each calibration standard had to be within ±20% of the nominal concentration at the LLOQ and within ±15% at all the other levels. At least 75% of the calibration standards should meet these criteria to demonstrate linearity.

#### 2.6.4 Accuracy and Precision

Accuracy and precision were evaluated by analyzing six QC replicates at four different analyte concentration levels: LLOQ, low QC (3× LLOQ), medium QC (30% calibration curve range), and high QC (75% ULOQ). Six replicates of each concentration level were processed the same day for intra‐run assay, and three runs in three different days over a period of 2 weeks were processed for the inter‐run assay. Accuracy had to be within 85%–115% of the nominal concentration and precision (%CV) of ±15%, except at the LLOQ, where accepted values were between 80% and 120% and ±20% for accuracy and precision, respectively.

#### 2.6.5 Carry‐Over

Carry‐over was assessed by analyzing blank samples after the calibration standard at the ULOQ and evaluating the presence of peaks at the retention time of ropivacaine or IS. For each standard curve, this was examined three times within one run and over five runs in total. Carry‐over was examined after peak integration in SciexOS software. Carry‐over was defined as minimal if the response at the blank samples was not greater than 20% of the analyte response at the LLOQ and not greater than 5% of the response for the IS.

#### 2.6.6 Stability

The stability of ropivacaine in dog plasma was assessed mimicking conditions during clinical application of the method. The stability of the analyte in the matrix was evaluated using low and high concentration QCs each in three replicates at different storage conditions. Freeze–thaw stability was assessed after 3 cycles of freezing and thawing, for which the samples were kept frozen at −20°C for at least 12 h between thawing. Bench‐top stability was evaluated by keeping the samples at room temperature for 5 h. Long‐term stability was evaluated for 10 days, both at −20°C and −80°C. The stability of ropivacaine in the processed samples was determined at 10°C by reanalysing the replicates of the four QC levels that were kept in the autosampler.

The mean concentration at each QC level should be within ±15% of the nominal concentration.

#### 2.6.7 Reinjection Reproducibility

Reinjection of a run that comprised calibration standards and six replicates of the LLOQ, low, middle, and high QC after storage in the autosampler at +10°C for 4 days was performed to evaluate the reproducibility of the method.

### 2.7 Complete Workflow Validation

#### 2.7.1 Repeatability

Repeatability of UHPLC–MS/MS method of the complete analysis including sample preparation and LC–MS analysis was established by analyzing blank plasma samples spiked with either a low (88 ng/mL) or high (1740 ng/mL) ropivacaine concentration. Sample preparation, which included RED, was performed on three different days within 12 days for *n* = 6 samples per each day. Sample preparation, LC–MS acquisition, and data evaluation were done separately for plasma and buffer samples to gain information about the whole sample preparation process. The accuracy at each concentration level had to be within ±15% of the nominal concentrations, and the precision (%CV) of the concentrations determined at each level had to be ±15%.

#### 2.7.2 Recovery

Determination of precipitation recovery as well as overall method recovery for the complete workflow including sample preparation and LC–MS analysis was performed according to Lamy et al. (2020). Three approaches (A, B, and C) were performed on each dog blank plasma or matrix‐free samples spiked at either 10 or 2000 ng/mL ropivacaine (Table 2). Precipitation recovery was defined by the ratio between the peak area obtained in Approach A “Plasma spiked before precipitation” and the peak area obtained in Approach B “Blank plasma matrix spiked after precipitation.” Overall recovery was defined as the ratio between the peak area obtained in Approach A “Plasma spiked before precipitation” and the peak area obtained in Approach C “Solvent spike.” We considered that the overall method recovery of the IS had to be ±15% of ropivacaine recovery.

**TABLE 2:** Overview on methods applied for determination of precipitation and overall method recovery.

Spike	Approach	Preparation	Standard curve
Preci spike	A	Matrix + Std → Precipitation → LC–MS	Matrix‐matched
Matrix spike	B	Matrix → Precipitation → + Std → LC–MS	Matrix‐matched
Solvent spike	C	H_2_O + Std → Precipitation → LC–MS	Solvent‐based

### 2.8 Study Samples Analysis

After obtaining approval from the National and Institutional Ethical Committees, the method was applied to analyze free and total ropivacaine instilled intraperitoneally in eight healthy dogs. Informed caregiver's written consent was obtained for all dogs enrolled. Dogs included were adults, female, with an actual body weight over 15 kg and a body condition score of 4–6/9, non‐pregnant, healthy, and that were scheduled to undergo ovariectomy or ovariohysterectomy. Health was assessed based on medical history, physical examination, hematology, and serum biochemistry. The dogs were anesthetized and randomized to be given 1 (Group R1; *n* = 4) or 3 mg/kg (Group R3; *n* = 4) ropivacaine, diluted with 0.9% NaCl to a total volume of 0.8 mL/kg. After ovariectomy/ovariohysterectomy, solution aliquots were instilled over the ovarian and uterine stumps. Jugular venous blood was sampled at −2, 5, 10, 15, 30, 45, 60, 120, and 240 min after instillation. Blood was collected in sterile syringes and immediately transferred to heparin‐containing tubes. Plasma was separated by centrifugation for 10 min at 1200*g* and then stored at −80°C until analyzed.

## 3 Results

### 3.1 Method Development

**FIGURE 2:**
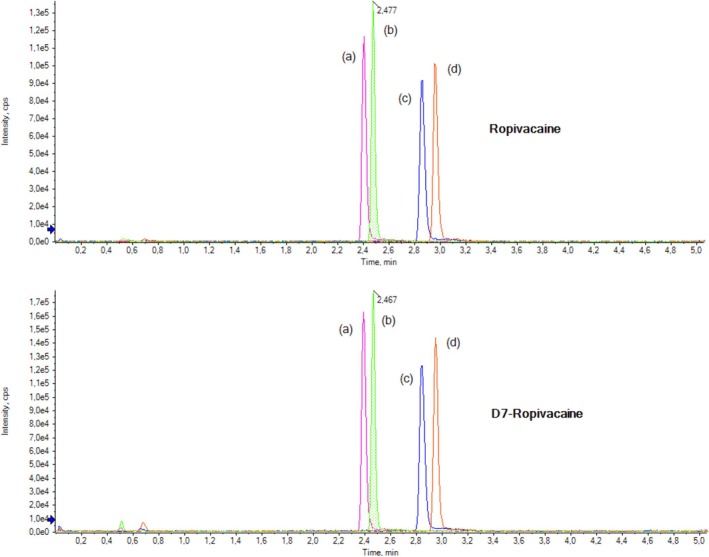
Chromatograms of ropivacaine standard (XIC m/z 275.1 ➔ 84.0) and the internal standard D7‐ropivacaine (XIC m/z 282.1 ➔ 85.0) comparing the Kinetex C18 and the Kinetex F5 UHPLC column using gradient elution with two different flow rates: Kinetex C18 (a) 0.4 mL/min and (c) 0.3 mL/min, Kinetex F5 (b) 0.4 mL/min and (d) 0.3 mL/min.

For the LC method development, two UHPLC columns were tested: a 1.7 μm Kinetex C18 100 Å and a 1.7 μm Kinetex F5 100 Å (both 100 × 2.1 mm inner diameter, Phenomenex Inc., Torrance, USA). Applying the gradient elution described in Section 2.5, two different flow rates (0.3 and 0.4 mL/min) were compared, as shown in Figure 2. Due to the narrower peak width and therefore larger peak height, the Kinetex F5 column at a flow rate of 0.4 mL/min was chosen for the final LC setup.

The two most intense transitions for ropivacaine were m/z 275.1 ➔ 126.0 and m/z 275.1 ➔ 84.0. The analysis of the standard curves resulted in poor linear regression for m/z 275.1 ➔ 126.0 within a concentration range between 0.1 and 1000 ng/mL (*R* ^2^ = 0.6108). Therefore, the next two most intense transitions, m/z 275.1 ➔ 84.0 and m/z 275.1 ➔ 98.2, were chosen as a quantifier and qualifier for ropivacaine, respectively. Accordingly, for the IS D7‐ropivacaine, the second most intense transition, m/z 282.1 → 85.1 Da, was chosen as a quantifier and m/z 282.1 → 105.1 as a qualifier (Table 1).

### 3.2 LC–MS Method Validation Procedure

#### 3.2.1 Selectivity and Specificity

**FIGURE 3:**
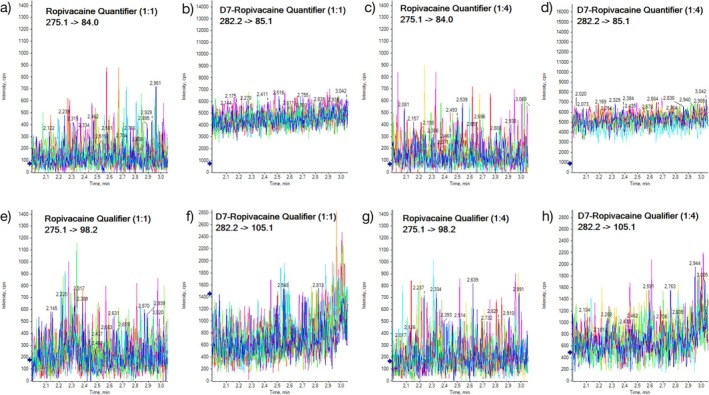
Overlaid blank chromatograms of six dogs: (a) quantifier ropivacaine 1:1 dilution, (b) quantifier IS in 1:1 dilution, (c) quantifier ropivacaine in 1:4 dilution, (d) quantifier IS in 1:4 dilution, (e) qualifier ropivacaine in 1:1 dilution, (f) qualifier IS in 1:1 dilution, (g) qualifier ropivacaine in 1:4 dilution, and (h) qualifier IS in 1:4 dilution. The lack of peaks at 2.4 min demonstrates the absence of any significant interfering components.

The plasma of six dogs without ropivacaine was evaluated and found to be free from potential interference substances. None of the samples had responses attributable to interfering components. Under the described chromatographic conditions, retention times were 2.4 min for ropivacaine and IS. No interfering peak of ropivacaine or the IS was observed in the 1:1 and 1:4 dilutions from drug‐free plasma samples (Figure 3).

#### 3.2.2 Matrix Effect

As shown in Table 3, the matrix effect was within the acceptable limits of accuracy (85%–115%) and precision (CV < 15%).

**TABLE 3:** Matrix effect assessed in dog plasma (*n* = 6) at low (LQC) and high (HQC) quality control levels (in 1:1 and 1:4 dilutions).

Dilution	QC level	Concentration (ng/mL) mean ± SD	Accuracy (%) mean ± SD	Precision (%) CV
1:1	LQC	0.14 ± 0.01	90.50 ± 5.50	6.10
	HQC	749.69 ± 44.88	100.00 ± 6.00	6.00
1:4	LQC	1.08 ± 0.02	107.80 ± 2.30	2.10
	HQC	821.76 ± 12.29	109.60 ± 1.60	1.50

Abbreviations: CV, coefficient of variation; SD, standard deviation.

#### 3.2.3 Calibration Curve and Range

**FIGURE 4:**
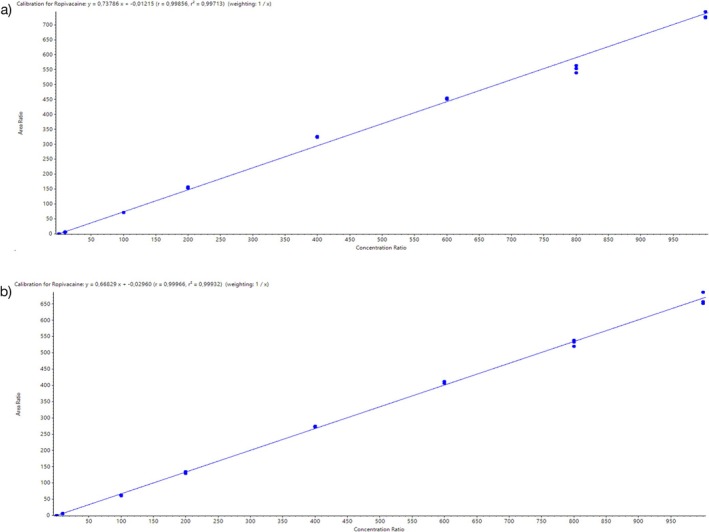
Standard curve in (a) 1:1 dilution (used for buffer fraction) and (b) 1:4 dilution (used for plasma fraction).

The calibration curves were linear over the concentration ranges of 0.05–1000 ng/mL in the 1:1 dilution (used for buffer fraction) and 0.075–1000 ng/mL for ropivacaine in the 1:4 dilution (used for plasma fraction). The mean coefficient of determination of the linear regression curves (*R* ^2^) was 0.9966 ± 0.0007 and 0.9991 ± 0.0002 (each *n* = 5) for the standard curve of the 1:1 and 1:4 dilutions, respectively. The linear regression (mean of five standard curves) was *y* = (0.727432 ± 0.012011) * *x* – (0.007252 ± 0.003736) for the 1:1 dilution, and *y* = (0.689414 ± 0.012898) * *x* – (0.042308 ± 0.014232) for the 1:4 dilution. Figure 4 shows an example graph of the standard curves for both dilutions.

The LLOQ was determined as the lowest analyzed concentration of ropivacaine with an accuracy of ±20% and showing the same ion ratio ropivacaine/IS as the higher standards. The ULOQ was determined as the highest ropivacaine concentration analyzed maintaining the linearity of the standard curve. LLOQ, ULOQ, and the corresponding QC levels are summarized in Table 4.

**TABLE 4:** Lower limit of quantification (LLOQ), upper limit of quantification (ULOQ), and quality control (QC) levels for determination of ropivacaine in rapid equilibrium dialysis (RED) buffer and plasma fractions.

Parameter		Matrix‐matched standard curve for RED buffer fraction (1:1) (ng/mL)	Matrix‐matched standard curve for RED plasma fraction (1:4) (ng/mL)
Lower limit of quantification	LLOQ	0.05	0.30
Low QC level	LQC	0.15	1.00
Medium QC level	MQC	300	300
High QC level	HQC	750	750
Upper limit of quantification	ULOQ	1000	1000

The accuracy of the back‐calculated concentrations of each calibration standard was within acceptance limits for all calibration curves.

#### 3.2.4 Accuracy and Precision

QC samples were analyzed at four concentrations to determine the accuracy and precision of this method. The results are presented in Table 5 and demonstrate that the developed method is accurate and precise for the evaluation of ropivacaine in dog plasma, over the tested concentration ranges. The intra‐run accuracy ranged from 90.50% to 113.30% and from 85.50% to 108.60% for the 1:1 and 1:4 dilutions, respectively. The inter‐run accuracy ranged from 91.80% to 106.80% and from 85.80% to 106.20% for the 1:1 and 1:4 dilutions, respectively.

**TABLE 5:** Intra‐ and inter‐run accuracy and precision (a) 1:1 (b) 1:4 dilutions.

(a)				
QC level 1:1	Parameter	Concentration (ng/mL) mean ± SD	Accuracy (%) mean ± SD	Precision (%) CV
LLOQ 0.05 ng/mL	Intra‐run 1	0.06 ± 0.00	113.30 ± 0.90	0.80
	Intra‐run 2	0.05 ± 0.00	100.90 ± 1.40	1.40
	Intra‐run 3	0.05 ± 0.00	106.20 ± 3.80	3.50
	Inter‐run	0.05 ± 0.00	106.80 ± 5.70	5.30
LQC 0.15 ng/mL	Intra‐run 1	0.14 ± 0.00	93.80 ± 1.20	1.30
	Intra‐run 2	0.14 ± 0.00	91.20 ± 1.70	1.80
	Intra‐run 3	0.14 ± 0.00	90.50 ± 0.60	0.70
	Inter‐run	0.14 ± 0.00	91.80 ± 1.90	2.00
MQC 300 ng/mL	Intra‐run 1	299.23 ± 3.15	99.70 ± 1.10	1.10
	Intra‐run 2	309.35 ± 2.38	103.10 ± 0.80	0.80
	Intra‐run 3	308.87 ± 3.90	103.00 ± 1.30	1.30
	Inter‐run	305.82 ± 5.66	101.90 ± 1.90	1.90
HQC 750 ng/mL	Intra‐run 1	736.58 ± 3.18	98.20 ± 0.40	0.40
	Intra‐run 2	757.70 ± 4.14	101.00 ± 0.60	0.50
	Intra‐run 3	752.09 ± 7.68	100.30 ± 1.00	1.00
	Inter‐run	748.79 ± 10.48	99.80 ± 1.40	1.40

(b)				
QC level 1:4	Parameter	Concentration (ng/mL) mean ± SD	Accuracy (%) mean ± SD	Precision (%) CV
LLOQ 0.30 ng/mL	Intra‐run 1	0.26 ± 0.00	87.50 ± 0.50	0.50
	Intra‐run 2	0.27 ± 0.01	88.70 ± 2.60	3.00
	Intra‐run 3	0.29 ± 0.01	97.00 ± 2.00	2.10
	Inter‐run	0.27 ± 0.01	91.10 ± 4.70	5.10
LQC 1.00 ng/mL	Intra‐run 1	0.86 ± 0.01	85.50 ± 0.70	0.80
	Intra‐run 2	0.86 ± 0.01	85.90 ± 0.50	0.60
	Intra‐run 3	0.86 ± 0.01	86.00 ± 0.60	0.80
	Inter‐run	0.86 ± 0.01	85.80 ± 0.60	0.70
MQC 300 ng/mL	Intra‐run 1	313.75 ± 3.06	104.60 ± 1.00	1.00
	Intra‐run 2	301.71 ± 2.80	100.60 ± 0.90	0.90
	Intra‐run 3	309.59 ± 2.24	103.20 ± 0.70	0.70
	Inter‐run	308.35 ± 5.74	102.80 ± 1.90	1.90
HQC 750 ng/mL	Intra‐run 1	814.26 ± 11.56	108.60 ± 1.50	1.40
	Intra‐run 2	778.78 ± 5.29	103.80 ± 0.70	0.70
	Intra‐run 3	795.73 ± 3.99	106.10 ± 0.50	0.50
	Inter‐run	796.25 ± 16.57	106.00 ± 2.00	2.10

Abbreviations: CV, coefficient of variation; HQC, high quality control; LLOQ, lower limit of quantification; LQC, low quality control; MQC, medium quality control; QC, quality control; SD, standard deviation.

#### 3.2.5 Carry‐Over

No carry‐over effect in the autosampler was detected up to concentrations of 400 ng/mL, for both dilutions of ropivacaine (1:1 and 1:4). However, sporadic carry‐over was detected in both qualifier and quantifier at concentrations higher than 400 ng/mL. It is therefore recommended to run blank samples after samples spiked with concentrations above 400 ng/mL. In the application of this method to the study samples, no interference due to carry‐over was expected because all samples had a ropivacaine concentration less than 400 ng/mL.

#### 3.2.6 Stability

Ropivacaine concentration in plasma samples (*n* = 3) spiked in two QC levels was evaluated for the ropivacaine concentration as well as accuracy and precision, under different storage conditions (Table 6). The mean accuracy ranged from 95.30% to 107.00% in the 1:1 dilution and from 103.50% to 112.60% in the 1:4 dilution. The stability of ropivacaine in plasma was demonstrated under the tested storage conditions.

**TABLE 6:** Stability of ropivacaine in dog plasma (*n* = 3) at low (LQC) and high (HQC) quality control levels (in 1:1 and 1:4 dilutions) under different storage conditions.

QC level	Storage condition	Concentration (ng/mL) mean ± SD	Accuracy (%) mean ± SD	Precision (%) CV
LQC 0.15 ng/mL 1:1	T0	0.15 ± 0.01	102.30 ± 5.60	5.50
	Benchtop	0.14 ± 0.01	96.60 ± 6.10	6.30
	Freeze/thaw	0.16 ± 0.00	104.00 ± 1.10	1.10
	T10/−20°C	0.15 ± 0.00	96.90 ± 1.60	1.60
	T10/−80°C	0.15 ± 0.01	98.20 ± 4.20	4.20
HQC 750.00 ng/mL 1:1	T0	739.40 ± 13.43	98.60 ± 1.80	1.80
	Benchtop	714.43 ± 16.77	95.30 ± 2.20	2.30
	Freeze/thaw	802.56 ± 12.17	107.00 ± 1.60	1.50
	T10/−20°C	748.73 ± 13.21	99.80 ± 1.80	1.80
	T10/−80°C	748.64 ± 16.68	99.80 ± 2.20	2.20
LQC 1.00 ng/mL 1:4	T0	1.02 ± 0.08	102.50 ± 8.00	7.80
	Benchtop	1.08 ± 0.04	108.20 ± 3.80	3.50
	Freeze/thaw	1.04 ± 0.03	104.20 ± 2.90	2.80
	T10/−20°C	1.03 ± 0.02	103.20 ± 2.50	2.40
	T10/−80°C	1.06 ± 0.06	106.00 ± 6.00	5.60
HQC 750.00 ng/mL 1:4	T0	849.5 ± 7.8	113.30 ± 1.00	0.90
	Benchtop	836.59 ± 12.27	111.50 ± 1.60	1.50
	Freeze/thaw	844.87 ± 3.03	112.60 ± 0.40	0.40
	T10/−20°C	810.93 ± 12.3	108.10 ± 1.60	1.50
	T10/−80°C	833.52 ± 7.46	111.10 ± 1.00	0.90

Abbreviations: CV, coefficient of variation; HQC, high quality control; LQC, low quality control; QC, quality control; SD, standard deviation.

#### 3.2.7 Reinjection Reproducibility

Reinjection reproducibility is shown in Table 7. Accuracy of the reinjected samples after storage in the autosampler was comparable to the first injection of the same samples: for the 1:1 dilution, 93.80%–113.30% (first run) vs. 90.10%–104.80% (reinjection); for the 1:4 dilution, 85.50%–108.60% (first run) vs. 85.10%–102.30% (reinjection). This shows that storage of the processed samples at +10°C for 4 days did not affect the ropivacaine concentration.

**TABLE 7:** Accuracy and precision of reinjected samples at 1:1 and 1:4 dilutions.

Dilution	QC level	Concentration (ng/mL) mean ± SD	Accuracy (%) mean ± SD	Precision (%) CV
1:1 dilution	LLOQ 0.05 ng/mL	0.05 ± 0.00	104.80 ± 3.40	3.20
	LQC 0.15 ng/mL	0.14 ± 0.00	90.10 ± 1.20	1.40
	MQC 300.00 ng/mL	320.87 ± 1.69	103.00 ± 0.90	0.90
	HQC 750.00 ng/mL	827.66 ± 7.32	99.00 ± 0.50	0.50
1:4 dilution	LLOQ 0.30 ng/mL	0.30 ± 0.01	86.30 ± 0.50	0.60
	LQC 1.00 ng/mL	0.87 ± 0.01	85.10 ± 0.40	0.50
	MQC 300.00 ng/mL	302.72 ± 1.55	100.30 ± 1.10	1.10
	HQC 750.00 ng/mL	780.55 ± 11.17	102.30 ± 1.30	1.30

### 3.3 Complete Workflow Validation

#### 3.3.1 Repeatability

Table 8 summarizes the intra‐run and inter‐run accuracy and precision data at two concentration levels spiked into the plasma sample before RED and precipitation, for bound, free, and total ropivacaine in dog plasma. The presented data confirms that the developed method including RED, precipitation, and LC–MS analysis is highly accurate and precise in this range of measured concentrations.

**TABLE 8:** Accuracy and precision data at two concentration levels for bound, free, and total ropivacaine in dog plasma (back‐calculated plasma concentrations).

Plasma spike (*n* = 6)	Plasma protein bound (*n* = 6 each day)	Free (*n* = 6 each day)	Total (*n* = 18)	
Concentration (ng/mL) mean ± SD (CV%)	Concentration (ng/mL) mean ± SD (CV%)	Concentration (ng/mL) mean ± SD (CV%)	Concentration (ng/mL) mean ± SD (CV%)	Accuracy in % mean ± SD (CV%)
87.70 ± 4.80 (5.50%)	D1: 87.90 ± 8.60 (9.80%)	D1: 4.20 ± 0.30 (6.00%)	D1: 92.10 ± 8.80 (9.50%)	D1: 105.00 ± 10.00 (9.50%)
	D2: 86.40 ± 5.60 (6.50%)	D2: 4.40 ± 0.20 (5.60%)	D2: 90.80 ± 5.60 (6.20%)	D2: 103.50 ± 6.40 (6.20%)
	D3: 96.80 ± 6.30 (6.30%)	D3: 4.60 ± 0.40 (9.00%)	D3: 101.60 ± 6.60 (6.50%)	D3: 115.70 ± 7.50 (6.50%)
	Total: 90.30 ± 8.10 (8.90%)	Total: 4.40 ± 0.40 (7.50%)	Total: 94.80 ± 8.30 (8.70%)	Total: 108.10 ± 9.40 (8.70%)
1742.50 ± 156.40 (9.00%)	D1: 1505.80 ± 118.50 (7.90%)	D1: 86.90 ± 4.70 (5.40%)	D1: 1592.70 ± 121.00 (7.60%)	D1: 91.40 ± 6.90 (7.60%)
	D2: 1566.80 ± 109.60 (7.00%)	D2: 89.10 ± 2.80 (3.20%)	D2: 1655.80 ± 108.90 (6.60%)	D2: 95.00 ± 6.20 (6.60%)
	D3: 1640.10 ± 63.00 (3.80%)	D3: 88.70 ± 9.60 (10.80%)	D3: 1728.80 ± 66.10 (3.80%)	D3: 99.20 ± 3.80 (3.80%)
	Total: 1570.90 ± 109.60 (7.00%)	Total: 88.20 ± 6.10 (6.90%)	Total: 1659.10 ± 111.10 (6.70%)	Total: 95.20 ± 6.40 (6.70%)

*Note:* D1, D2, D3: intra‐run repeatability/total: inter‐run repeatability.

Abbreviations: CV, precision calculated as coefficient of variation; D1, Day 1; D2, Day 2; D3, Day 3; SD, standard deviation.

#### 3.3.2 Recovery

The results of the precipitation and overall recovery for the two concentration levels of the complete sample preparation including RED, precipitation, and LC–MS data analysis workflow are shown in Table 9. The overall recovery for the ratio ropivacaine to D7‐ropivacaine was 110.50 ± 10.80% and 96.60 ± 7.80% for 10 and 2000 ng/mL concentration levels, respectively. The recovery of ropivacaine to D7‐ropivacaine was therefore within ±15%, independently of the concentration level. This is in agreement with the European Medicines Agency (2022) guidelines, where it is stated that the recovery of the analyte does not need to be 100%, as long as the extent of recovery of the analyte and of the IS are consistent. Therefore, it can be concluded that sample loss during precipitation could be compensated by the use of D7‐ropivacaine as an IS.

**TABLE 9:** Precipitation and overall recovery for samples spiked at two concentration levels of ropivacaine (10 and 2000 ng/mL).

**Spike 10 ng/mL**	**Concentration**	**Spike (each *n* = 5)**	**Concentration (ng/mL) mean ± SD (CV%)**	**Accuracy (%) mean ± SD (CV%)**
		(A) Preci spike 10	9.00 ± 0.70 (7.90%)	90.00 ± 7.20 (7.90%)
		(B) Matrix spike 10	8.20 ± 0.80 (10.00%)	81.60 ± 8.10 (10.00%)
		(C) Solvent spike 10	8.70 ± 0.40 (4.80%)	86.60 ± 4.20 (4.80%)
	**Recovery**	**Peak area of**	**Precipitation recovery (%) mean ± SD (CV%)** ^**a**^	**Overall recovery (%) mean ± SD (CV%)** ^**b**^
		Ropivacaine	125.20 ± 13.70 (11.00%)	103.90 ± 6.00 (5.80%)
		D7‐ropivacaine	114.00 ± 5.30 (4.60%)	94.50 ± 7.60 (8.10%)
		**Ratio ropivacaine/IS**	**110.10 ± 13.20 (12.00%)**	**110.50 ± 10.80 (9.80%)**
**Spike 2000 ng/mL**	**Concentration**	**Spike (each *n* = 5)**	**Concentration (ng/mL) mean ± SD (CV%)**	**Accuracy (%) mean ± SD (CV%)**
		(A) Preci spike 2000	2100.70 ± 121.50 (5.80%)	105.00 ± 6.10 (5.80%)
		(B) Matrix spike 2000	1849.50 ± 144.50 (7.80%)	92.50 ± 7.20 (7.80%)
		(C) Solvent spike 2000	1991.20 ± 69.90 (3.50%)	99.60 ± 3.50 (3.50%)
	**Recovery**	**Peak area of**	**Precipitation recovery (%) mean ± SD (CV%)** ^**a**^	**Overall recovery (%) mean ± SD (CV%)** ^**b**^
		Ropivacaine	144.60 ± 25.10 (17.40%)	99.70 ± 13.80 (13.80%)
		D7‐ropivacaine	126.60 ± 15.80 (12.50%)	103.50 ± 14.20 (13.70%)
		**Ratio ropivacaine/IS**	**114.20 ± 14.30 (12.50%)**	**96.60 ± 7.80 (8.10%)**

^a^ Precipitation recovery: Peak area “(A) Plasma spiked before precipitation” / Peak area “(B) Blank plasma matrix spiked after precipitation”.

^b^ Overall recovery: Peak area “(A) Plasma spiked before precipitation” / Peak area “(C) Solvent spike”.

### 3.4 Study Samples Analysis

**FIGURE 5:**
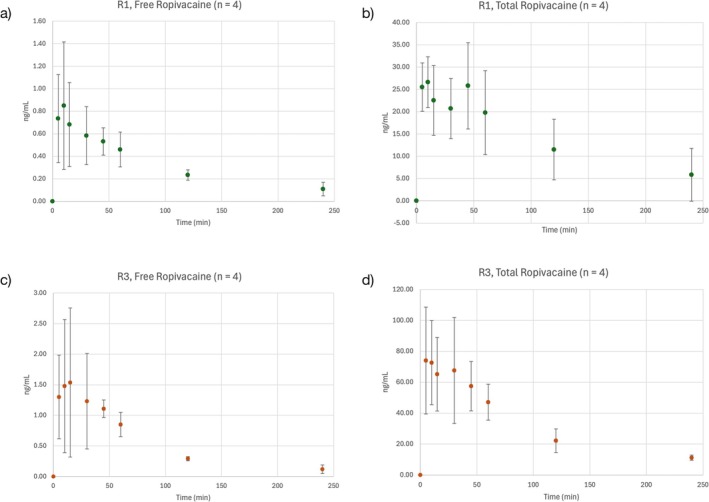
Mean concentrations of ropivacaine and their standard deviation over time analyzed in (a) buffer and (b) plasma fractions of study samples after intraperitoneal instillation of 1 mg/kg ropivacaine to anesthetized dogs (R1, *n* = 4), and mean concentrations of ropivacaine and their standard deviation over time in (c) buffer and (d) plasma fractions of study samples after intraperitoneal instillation of 3 mg/kg ropivacaine to anesthetized dogs (R3, *n* = 4).

The applicability of the method was demonstrated in a study after intraperitoneal instillation of 1 and 3 mg/kg ropivacaine in dogs. The mean plasma measured concentrations over time profiles of free and total ropivacaine are illustrated in Figure 5. The mean plasma analyzed concentrations of free and total ropivacaine were back calculated to the original plasma concentrations using eqs. 1 and 2 (Supporting Information S1: Appendix A). The mean (±SD) original peak plasma concentrations of free and total ropivacaine were 20.57 ± 13.73 ng/mL and 234.29 ± 49.82 ng/mL, and 37.23 ± 29.49 ng/mL and 661.25 ± 311.32 ng/mL for Group R1 and Group R3, respectively.

## 4 Discussion

A quantitative LC/MS method to determine free and total ropivacaine plasma concentration in dogs has been developed and validated with respect to selectivity, specificity, matrix effect, calibration curve and range, accuracy and precision, carry‐over, stability, and reinjection reproducibility. This is the first report of a highly sensitive and reliable method that can be further applied to evaluate free and total ropivacaine pharmacokinetics in dogs.

Local anesthetics are protein‐bound drugs, and the extent to which this occurs in the body has significant pharmacokinetic and pharmacodynamic implications. Indeed, the free fraction of the drug is responsible for reaching the target site and exerting its effect, and therefore knowing the amount of free drug is important to determine a safe dosage (Abbas et al. 2013). Among the several existing methods to determine the fraction of free and total protein‐bound drugs such as ultrafiltration and microdialysis, equilibrium dialysis has long been considered the gold standard approach (Banker and Clark 2008; Stumpe et al. 2000). Equilibrium dialysis using RED device inserts is a currently recommended technique tested in humans (Abbas et al. 2013; Lamy et al. 2020) and therefore we used it to achieve separation between free and protein‐bound ropivacaine in dog plasma.

The presented LC–MS method was fully validated according to the ICH M10 guidelines (2022). These guidelines further recommend that the samples for method validation be prepared in the same way anticipated for study samples. Therefore, besides the LC–MS method validation, repeatability and recovery validation of the complete workflow, including the RED step, was additionally performed. This is a distinct advantage in comparison to previous studies that determined free and total ropivacaine concentrations in plasma (Lamy et al. 2020; Mathieu et al. 2006), which increases the robustness of our method.

As in general, dilution of ropivacaine occurs because of protein precipitation during sample preparation and only 5% of ropivacaine is found to be free in the plasma; the final concentrations of ropivacaine in the buffer and the plasma fractions differ. In the lower spike concentration samples (88 ng/mL) used for complete workflow repeatability validation, the ropivacaine concentration in buffer analyzed by LC–MS was finally near the LLOQ at only 0.08 ng/mL, resulting in 4.4 ng/mL after recalculation to the original concentration in the buffer sample. Likewise, the higher spike concentration (1740 ng/mL) representing 95% of protein‐bound ropivacaine plus the small percentage of free ropivacaine in the plasma fraction after RED resulted in an analyzed ropivacaine concentration of 200 ng/mL. To compensate for this, separate evaluation of buffer and plasma fraction concentrations was established within the presented method. The very low concentration in buffer was analyzed undiluted in the matrix after precipitation, whereas for analysis of the higher ropivacaine concentrations in the plasma fraction, the sample was diluted 1:4 with water in order to avoid potential exceeding of the linear range of the calibration curve. Additionally, in order to minimize matrix interference, two separate standard curves matching the sample conditions were set up and used for further data evaluation. To our knowledge, this is the first RED approach that includes the use of two standard curves for the assessment of both total and free ropivacaine fractions.

This method was proven to be highly sensitive as the LLOD was as low as 0.05 ng/mL for the quantitation of free ropivacaine in the buffer fraction, which is useful to determine plasma concentrations of free ropivacaine in further clinical studies. The transitions chosen as a quantifier for ropivacaine in this method were m/z 275.11 ➔ 84.00, which differ from the transitions chosen in other studies (Butiulca et al. 2023; Lamy et al. 2020). The method presented here aimed to compensate for potential matrix effects, not only by using D7‐ropivacaine as an IS but also by using matrix‐matched standard curves in two different dilutions: for the analysis of the very low concentrated free ropivacaine fractions, the standard curve was diluted only in blank plasma after precipitation, whereas for the higher concentrated plasma fraction, samples and standards were diluted 1:4 with water.

Although LC–MS methods to determine the concentration of ropivacaine and other local anesthetics in dog plasma have been briefly described in pharmacokinetic or clinical studies (Abimussi et al. 2014; Adami et al. 2012; Salvo et al. 2015; Wilcke et al. 1983), no method validation to determine free and total ropivacaine concentration in dog plasma has yet been reported. Distinct advantages of the present method include the quantification of both free and total ropivacaine, the selection of adequate MRM transitions, very high sensitivity, a clinically relevant range of the calibration curve, the use of D7‐ropivacaine as an IS, the inclusion of RED during sample preparation, the compensation for different concentration levels of free and protein‐bound ropivacaine, and potential matrix effects using two standard curves.

## 5 Clinical Applicability

Ropivacaine is one of the most commonly used local anesthetics both in human and veterinary medicine (El‐Boghdadly et al. 2018; Grubb and Lobprise 2020). In dogs, several studies have evaluated the analgesic efficacy of intraperitoneally instilled ropivacaine (Brioschi et al. 2023; Kazmir‐Lysak et al. 2023; Lambertini et al. 2018), and other studies have characterized the pharmacokinetics of ropivacaine administered by other routes (Abimussi et al. 2014; Adami et al. 2012; Arthur et al. 1988; Morgaz et al. 2021). However, the pharmacokinetics of ropivacaine instilled intraperitoneally have not been yet studied in dogs. Furthermore, to date, no method validation to measure free and total ropivacaine in dog plasma has been yet reported. Because of its high sensitivity, this method could help bridge the gap between analytical and clinical regional anesthesia research and serve as a basis to better characterize the pharmacokinetic profile of free and total ropivacaine in dogs.

## 6 Conclusion

A highly sensitive and reliable UHPLC–MS/MS method was developed and validated according to the ICH M10 guidelines for the determination of free and total ropivacaine in dog plasma samples. The overall performance of the method was proven to be linear, accurate, and precise as well as repeatable with an adequate recovery.

## Conflicts of Interest

The authors declare no conflicts of interest.

## Supporting information


**Data S1:** Supporting information.

## Data Availability

The data that support the findings of this study are available from the corresponding author upon reasonable request.
