# Supplementary material for: A Method for Analysis of Free and Total Ropivacaine in Dog Plasma Using UHPLC–MS/MS
Source: Biomed Chromatogr. 2025 Sep 3;39(10):e70214. doi: 10.1002/bmc.70214 (PMC12408134; doi:10.1002/bmc.70214)
Supplement: Supplementary file 1 — Data S1: Supporting information. [file BMC-39-e70214-s001.docx]

**Appendix A. Supplementary material**

Calculation of free and protein-bound ropivacaine concentration was performed according to the formulas given below.

Description of used variables:

$C_{PlasmaFree}$ Concentration of free ropivacaine in the original plasma sample (ng/mL)

$C_{PlasmaBound}$ Concentration of protein-bound ropivacaine in the original plasma sample (ng/mL)

$C_{BufferLCMS}$ Concentration of ropivacaine in the RED PBS buffer fraction according to LC-MS analysis (ng/mL)

$C_{PlasmaLCMS}$ Concentration of ropivacaine in the RED plasma fraction according to LC-MS analysis (ng/mL)

$\sum{\mu l}_{TotalPrec}$ Sum of all components (µl) added for protein precipitation

${\mu l}_{Plasma}$ Total amount of plasma sample (µl) added to the RED chamber

${\mu l}_{PBS}$ Total amount of PBS buffer (µl) added to the RED chamber

${\mu l}_{PrecBuffer}$ Amount of buffer sample (µl) from the buffer chamber used for precipitation after rapid equilibrium dialysis

${\mu l}_{PrecPlasma}$ Amount of plasma sample (µl) from the plasma chamber used for precipitation after rapid equilibrium dialysis

${\mu l}_{ACN}$ Amount of acetonitrile (µl) added for protein precipitation

${\mu l}_{FA}$ Amount of 0.1% formic acid (µl) added for protein precipitation

${\mu l}_{IS}$ Amount of internal standard (µl) added for protein precipitation

**Eq. A1:**

Calculation of free ropivacaine concentration: $C_{PlasmaFree}$

$$C_{PlasmaFree}=\frac{C_{BufferLCMS}*\sum{\mu l}_{TotalPrec}*({\mu l}_{Plasma}+{\mu l}_{PBS})}{{\mu l}_{PrecBuffer}* {\mu l}_{Plasma}}$$

$$\sum{\mu l}_{TotalPrecBuffer}={\mu l}_{PrecBuffer}+ {\mu l}_{PrecBuffer}+{\mu l}_{ACN}+ {\mu l}_{Formic acid} + {\mu l}_{IS}$$

**Eq. A2:**

Calculation of protein-bound ropivacaine concentration: $C_{PlasmaBound}$

$$C_{PlasmaBound}=\frac{(\frac{C_{PlasmaLCMS}*\sum{\mu l}_{TotalPrecPlasma}}{1000}-\frac{C_{BufferLCMS}*\sum{\mu l}_{TotalPrecBuffer}}{1000})*1000}{{\mu l}_{PrecPlasma}}$$

$$\sum{\mu l}_{TotalPrecPlasma}={\mu l}_{PrecPlasma}+ {\mu l}_{PrecPlasma}+{\mu l}_{ACN}+ {\mu l}_{Formic acid} + {\mu l}_{IS}$$
